# Community-based mass treatment with azithromycin for the elimination of yaws in Ghana—Results of a pilot study

**DOI:** 10.1371/journal.pntd.0006303

**Published:** 2018-03-22

**Authors:** Abdul Aziz Abdulai, Patrick Agana-Nsiire, Frank Biney, Cynthia Kwakye-Maclean, Sardick Kyei-Faried, Kwame Amponsa-Achiano, Shirley Victoria Simpson, George Bonsu, Sally-Ann Ohene, William Kwabena Ampofo, Yaw Adu-Sarkodie, Kennedy Kwasi Addo, Kai-Hua Chi, Damien Danavall, Cheng Y. Chen, Allan Pillay, Sergi Sanz, Ye Tun, Oriol Mitjà, Kingsley Bampoe Asiedu, Ronald C. Ballard

**Affiliations:** 1 West Akim District Health Administration, Ghana Health Service, Asamankese, Ghana; 2 National Yaws Eradication Programme, Ghana Health Service, Accra, Ghana; 3 District Hospital Laboratory, Ghana Health Service, Asamankese, Ghana; 4 Disease Control Department, Ghana Health Service, Accra, Ghana; 5 Expanded Programme on Immunization, Ghana Health Service, Accra, Ghana; 6 Noguchi Memorial Institute for Medical Research, University of Ghana, Accra, Ghana; 7 World Health Organization Country Office, Accra, Ghana; 8 School of Medical Sciences, Kwame Nkrumah University of Science and Technology, Kumasi, Ghana; 9 Laboratory Reference and Research Branch, Division of STD Prevention, Centers for Disease Control and Prevention, Atlanta, Georgia, United States of America; 10 Barcelona Institute for Global Health, Hospital Clinic – University of Barcelona, Barcelona, Spain; 11 Center for Global Health, Centers of Disease Control and Prevention, Atlanta, Georgia, United States of America; 12 Department of Community Health, Lihir Medical Centre, Lihir Island, Papua, New Guinea; 13 Department of Control of Neglected Tropical Diseases, World Health Organization, Geneva, Switzerland; Johns Hopkins Bloomberg School of Public Health, UNITED STATES

## Abstract

**Introduction:**

The WHO yaws eradication strategy consists of one round of total community treatment (TCT) of single-dose azithromycin with coverage of > 90%.The efficacy of the strategy to reduce the levels on infection has been demonstrated previously in isolated island communities in the Pacific region. We aimed to determine the efficacy of a single round of TCT with azithromycin to achieve a decrease in yaws prevalence in communities that are endemic for yaws and surrounded by other yaws-endemic areas.

**Methods:**

Surveys for yaws seroprevalence and prevalence of skin lesions were conducted among schoolchildren aged 5–15 years before and one year after the TCT intervention in the Abamkrom sub-district of Ghana. We used a cluster design with the schools as the primary sampling unit. Among 20 eligible primary schools in the sub district, 10 were assigned to the baseline survey and 10 to the post-TCT survey. The field teams conducted a physical examination for skin lesions and a dual point-of-care immunoassay for non-treponemal and treponemal antibodies of all children present at the time of the visit. We also undertook surveys with non-probabilistic sampling to collect lesion swabs for etiology and macrolide resistance assessment.

**Results:**

At baseline 14,548 (89%) of 16,287 population in the sub-district received treatment during TCT. Following one round of TCT, the prevalence of dual seropositivity among all children decreased from 10.9% (103/943) pre-TCT to 2.2% (27/1211) post-TCT (OR 0.19; 95%CI 0.09–0.37). The prevalence of serologically confirmed skin lesions consistent with active yaws was reduced from 5.7% (54/943) pre-TCT to 0.6% (7/1211) post-TCT (OR 0.10; 95% CI 0.25–0.35). No evidence of resistance to macrolides against *Treponema pallidum* subsp. *pertenue* was seen.

**Discussion:**

A single round of high coverage TCT with azithromycin in a yaws affected sub-district adjoining other endemic areas is effective in reducing the prevalence of seropositive children and the prevalence of early skin lesions consistent with yaws one year following the intervention. These results suggest that national yaws eradication programmes may plan the gradual expansion of mass treatment interventions without high short-term risk of reintroduction of infection from contiguous untreated endemic areas.

## Author summary

In this study, we provided a single round of total community treatment (TCT) with azithromycin to the population of a sub-district in Ghana (16,287 people) that is endemic for yaws and surrounded by other yaws-endemic communities to determine whether a sustained decrease in yaws prevalence could be achieved up to one year after the intervention. The efficacy of TCT was assessed by performing a clinical evaluation and serological testing of any yaws-like lesions found as well as serological screening of asymptomatic schoolchildren aged 5–15 years pre-TCT and at one year post-TCT. The results indicate that after a single round of high coverage TCT (89%) with azithromycin, the prevalence of active and latent yaws was significantly reduced. We also found that the use of a dual point-of-care immunoassay to detect non-treponemal and treponemal antibodies among school-going children is a practical alternative to laboratory-based serological testing to evaluate the effectiveness of yaws interventions in resource-poor settings.

## Introduction

Yaws is a chronic, relapsing, neglected tropical disease caused by *Treponema pallidum* spp. *pertenue*, a spirochaete closely related to that which causes syphilis [1]. The disease is currently reported from 13 of the 85 countries previously considered endemic in the 1950s, with an estimated 89 million people living in the affected districts [2]. Yaws is usually acquired by children in impoverished communities in tropical and subtropical countries when traumatized skin comes into contact with another person’s early infectious lesion exudate, often during play. The disease affects mostly children aged under 15 years. The frequency of infection appears to be higher in boys than in girls [3].

Primary yaws lesions develop at the site of initial inoculation after an incubation period of 9–90 days. These lesions are initially papules, which can develop into papillomata and eventually ulcerate, and are most frequently found on the lower legs and ankles and, less frequently, on the skin of the upper limbs and elsewhere on the body. If left untreated, the disease may progress to the secondary stage, which is characterized by multiple skin lesions as well as osteitis and periostitis of the bones underlying the skin lesions. Untreated disease may spontaneously resolve clinically and enter a period of latency prior to the development of non-infectious gummas of the skin, cartilage and bone, resulting in the destructive, often disfiguring, lesions of late yaws [4].

During the 1950s and 1960s, the World Health Organization (WHO) and the United Nations Children’s Fund (UNICEF) led a global campaign to eradicate the disease by providing mass treatment to affected communities using single intramuscular injections of long-acting penicillin. The strategy was based on the need to screen at least 90% of the population, treat the entire reservoir of treponemal infection (including those with clinical disease, latent infection and contacts) and to perform periodic surveys at 6–12 months to identify and treat missed, new and imported cases [5]. The criteria for eradication were defined by the WHO Expert Committee on Venereal Infections and Treponematoses in 1960 as the absence of an indigenous infectious case in the population for three consecutive years and absence of any new seroreactor aged under 5 years [6]. The implementation of the global yaws eradication programme between 1952–1964 reduced the prevalence of infection by approximately 95% (from 50 million to 2.5 million cases) worldwide, indicating that the mass treatment approach using benzathine penicillin was highly successful [7]. In some countries such as Haiti and Nigeria, experience showed that one round of mass treatment with coverage of > 90% significantly reduced the prevalence of infectious cases by approximately 98% within 12 months [8, 9]. Despite the success of the campaign, the ultimate goal of eradication could not be achieved owing to several factors including the failure of many countries to adequately integrate active surveillance activities into the local health system after the mass treatment campaigns ended, complacency, limited resources, lack of political will and many new competing health priorities including the shifting of the dedicated mobile teams for yaws eradication to deal with diseases such as smallpox and cholera [5]. This situation led to the resurgence of yaws in several countries in the late 1970s, prompting the World Health Assembly to adopt resolution WHA31.58 [10] which called on countries to take the necessary measures to interrupt transmission at the earliest possible time. Despite the possibility that yaws transmission has ceased in a number of countries that were endemic in the 1950s but not confirmed, only India has recently been formally certified by WHO as yaws-free [11]. The disease still remains endemic in many countries in West and Central Africa, South-East Asia and the Western Pacific [12].

In 2012, the finding that a single oral dose of azithromycin was as effective as injectable penicillin for the treatment of yaws [13] prompted WHO to revisit the global eradication of the disease. In 2012, WHO published a roadmap on neglected tropical diseases that targeted yaws eradication by 2020 [14]. In the same year, WHO devised a new yaws eradication strategy (the Morges Strategy) [15]. The strategy recommends total community treatment (TCT, equivalent to mass treatment for other neglected tropical diseases) of affected communities with single doses of azithromycin followed by ongoing active surveillance, case-finding and treatment of missed, new and imported cases and their contacts (household, neighborhood and school playmates) through a health system approach. Depending on the initial treatment coverage and accessibility to the endemic communities, the strategy recommends repeat surveys to identify and treat any new infectious cases or in response to localized outbreaks (using total targeted treatment, TTT). The feasibility of global yaws eradication and the progress made in implementing the Morges Strategy have previously been reported [16, 17].

In 2015, the first empirical data of the impact of mass treatment with azithromycin on disease transmission became available. A study carried out in Lihir, Papua New Guinea (PNG) demonstrated that mass treatment with azithromycin to the population of yaws-endemic island communities resulted in a significant decrease in the prevalence of clinically early yaws lesions and a decrease in reactive serological markers for the disease [17]. Similar findings have emerged from a study in the Solomon Islands that evaluated the secondary benefits of mass treatment of trachoma using 20 mg/kg azithromycin on the prevalence of yaws [18]. However, despite the encouraging results obtained in these countries, focused mass treatment campaigns are, theoretically, more likely to succeed in isolated island communities where the risk of reintroduction of infection in the treated population from untreated adjoining communities is less likely. Since the disease is also endemic in many countries with affected communities spread over a contiguous land mass, it is also important to determine whether high coverage with azithromycin mass treatment in a defined area can result in a sustained decrease in infectious yaws up to one year after the intervention. This is also important, since the resources available to yaws eradication programmes in many countries are limited, resulting in an inevitable delay or progressive expansion of the implementation of the programme to adjoining endemic areas that could act as a source of re-infection for the initial target communities.

In this study, we aimed to assess the impact of a single round of TCT with azithromycin using two markers of infection among school-going children in the target communities before and one year after the intervention. We measured the prevalence of dually seropositive for non-treponemal and treponemal serological markers, and the prevalence of active yaws-like lesions among children. A secondary objective was to establish the etiology of active yaws-like lesions among schoolchildren using sensitive molecular techniques and to assess the occurrence of mutations associated with azithromycin resistance among *T*. *pallidum* spp. *pertenue* positive lesions in the local population.

## Methods

The study protocol was approved by the ethics committee of the Ghana Health Service, Ministry of Health (GHS-ERC-05/01/13). Written informed consent was obtained from parents and where they were unable to provide consent, teachers provided written consent on their behalf, which is normal practice for mass treatments campaigns in Ghana. Prior to the initiation of mass treatment with azithromycin, information sessions were held with the regional directorate of health services, the district political authorities and communities about the study in order to gain their support. The local radio was used to inform the entire district about the mass treatment campaign. Health workers and village volunteers distributed the WHO yaws picture booklets [19] to households and schools as part of the social mobilization effort.

### Study area and population

**Fig 1:**
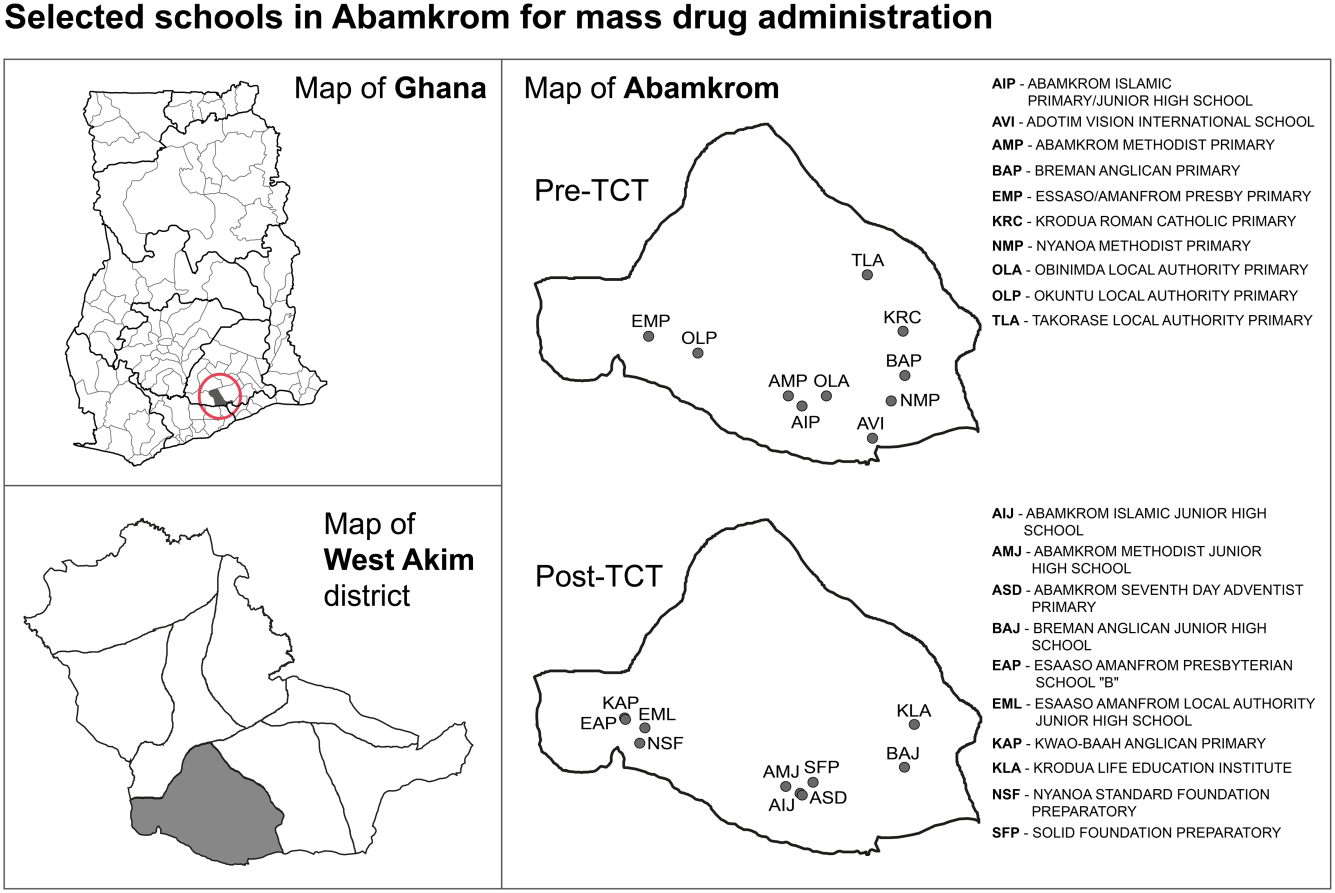
Maps showing position of Abamkrom sub-district within the West Akim district in Ghana and geographical distribution of schools sampled pre- and post-TCT.

We conducted a prospective observational study in the Abamkrom sub-district, West Akim district of the Eastern Region of Ghana between October 2013 and December 2014. The sub-district is highly endemic for yaws (Fig 1) [20]. The total population of the sub-district in mid-2013 was estimated to be 16,287 people, i.e. 7.7% of the population of the district as a whole (212 282 people). The eligible population for mass treatment was everyone older than 6 months of age, excluding pregnant women (as decided by the local study team), 15,310 people in total. The sub-district has 5 health centres, 24 primary schools and 36 communities. The total population of children 5–15 years registered in primary schools that were targeted for impact assessment surveys was 2,909 children. Each community has a village health volunteer who is responsible for monitoring and reporting health events. These volunteers are used for mass drug administration for other neglected tropical diseases. The total eligible population (aged ≥ 6 months) living in the 36 communities was targeted to receive mass treatment with azithromycin as part of the study. The school attendance rate in the sub-district among children aged 5–15 years has been estimated to be 47.6%.

### Mass azithromycin treatment

Prior to the implementation of the study, the health workers and village volunteers were trained on the objectives of the study, recognition of yaws-like lesions, implementation and data collection tools. The TCT programme was conducted by 13 teams of two trained volunteers drawn from affected communities who were supervised by local health-care workers or members of the national yaws eradication programme. During a five-day period in November–December 2013, the teams offered azithromycin tablets (purchased by WHO from Medopharm, Chennai, India) at a single oral dose of 30 mg/kg per body weight (maximum 2 g) to all members of the 36 targeted communities aged 6 months and above at no cost to the participants. The tablets (500g strength) were administered to the eligible population according to age as described in the WHO Morges Strategy document [15]. For children aged under 6 years, the tablets were crushed and mixed with water. The volunteers directly observed treatment of participants, maintained tally sheets; they marked the fingers of treated participants with indelible ink to document the administration of the medicines, and observed participants for approximately one hour after ingestion of the medication, reporting any adverse events that could be related to azithromycin treatment. The supervisors collected the tally sheets daily and followed up any adverse drug reactions reported by volunteers. TCT coverage rates were calculated using the number of persons treated according to the tally sheets divided by the total population.

### School and participants selection

We used cluster sampling with individual schools as the cluster unit. Eligibility for inclusion was met by 20 primary schools with a population larger than 100 children among 24 schools located in the sub-district. The schools were randomly assigned to either the pre- or post- TCT evaluation surveys. Therefore, the sets of schools for pre- and post- TCT surveys were mutually exclusive and schools could not be chosen repeatedly for more than one survey. Every child present at the time of our visit to the schools selected was invited to participate in the study.

### Procedures

The primary outcome was prevalence of *sero-positivity*, defined as dually non-treponemal and treponemal antibody positivity using a point-of-care test among symptomatic or asymptomatic children. Secondary outcomes, were the prevalence of *suspected yaws*, defined as a child with a history of residence in an endemic area who presented with clinically active (visible) yaws-like lesions, and the prevalence of *serologically-confirmed yaws*, defined as a suspected case with a dually-positive serological test result for non-treponemal and treponemal antibodies.

**Fig 2:**
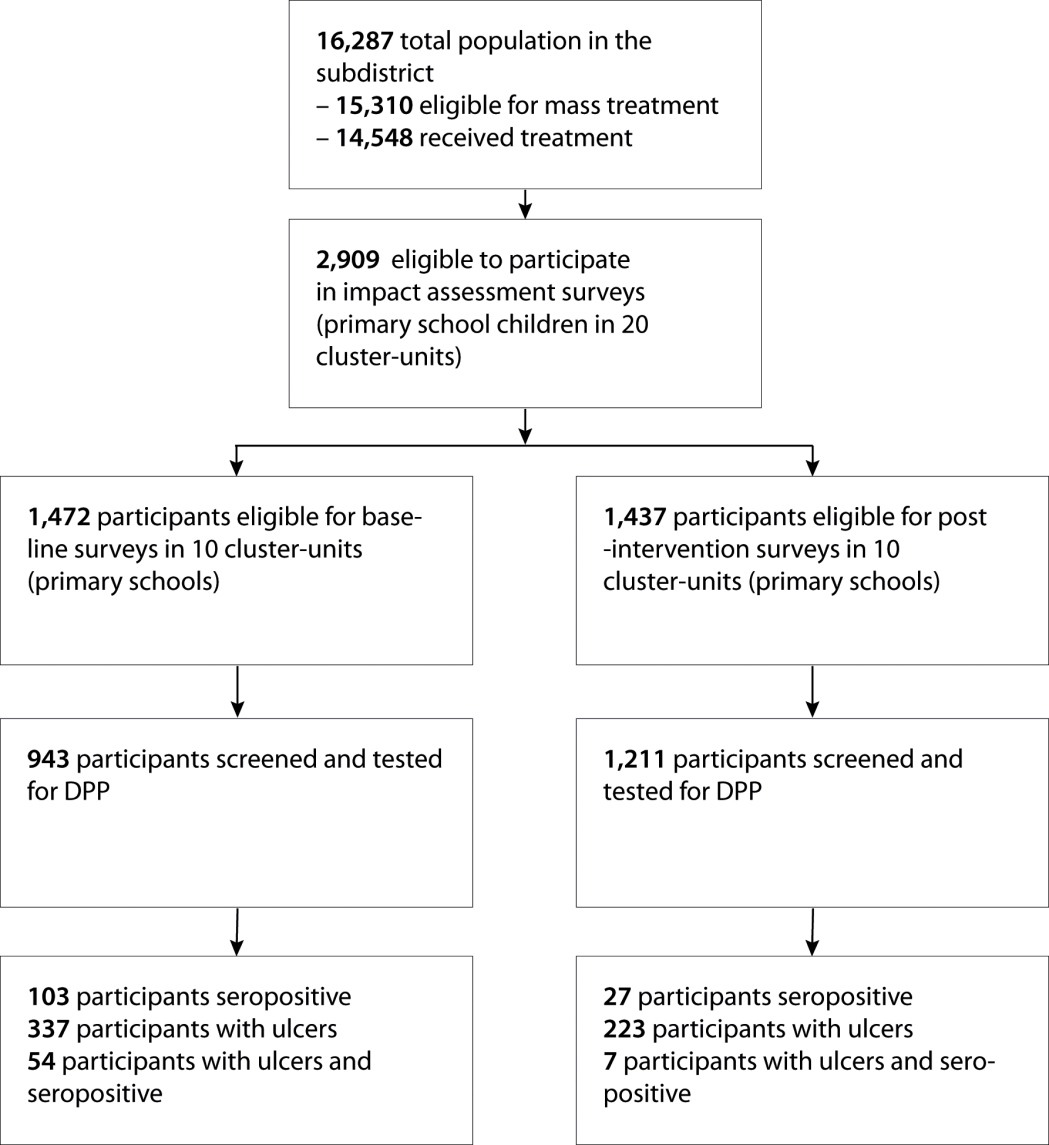
Flowchart of school and pupil selection.

In October 2013, before the mass treatment campaign (November–December 2013), we conducted a baseline assessment survey in 10 randomly selected primary schools and every child present, 943 children in total, was enrolled. One year after the intervention (November–December 2014), every child from a further group of 10 randomly selected schools, 1211 children in total, were selected for an identical post-intervention assessment of impact survey. Fig 1 shows the geographical distribution of these schools within the sub-district and Fig 2 the flowchart of the study design.

During both assessments, all children were examined clinically for skin lesions consistent with early infectious yaws (i.e. skin papilloma, chronic solitary or multiple skin ulcerations) and a specimen of capillary blood was collected from all, symptomatic and asymptomatic, participants to perform a point-of-care immunoassay for antibody to yaws infection. All children with active yaws-like lesions detected during either the initial or post-TCT assessments or those who were asymptomatic but had a dually non-treponemal and treponemal antibody-positive result on initial field screening were treated with a single dose of azithromycin (30 mg/kg) and followed up for adverse events.

### Serological testing

A point-of-care immunoassay, developed for the serological diagnosis of syphilis, that can simultaneously detect both non-treponemal and treponemal antibodies (DPP Syphilis Screen and Confirm Assay, Chembio Diagnostic Systems, Medford, NY, USA) [21–23] was used to test 10 μl samples of capillary blood obtained by finger prick from each of the schoolchildren who were included in the selected pre- and post-TCT schools. In each case, the child’s finger was cleaned with an alcohol swab, the skin punctured with a lancet and the capillary blood collected in a pre-graduated micropipette supplied in the test kit. Thereafter the test was performed directly in the field according to the manufacturer’s instructions.

### Surveys to determine the etiology of active yaws-like lesions and mutations for azithromycin resistance

We conducted clinical surveys to collect lesion swabs for PCR testing before and one year after TCT. Due to the low number of *T*. *pallidum* PCR positive cases detected in Abamkrom sub-district before the intervention, we decided to extend the baseline study to the entire West Akim district (8 sub-districts, 212 282 people). We had 8 teams, one in each sub-district, that examined the skin of schoolchildren aged less than 15 years of age. The sampling for this study was non-probabilistic; teams visited enough schools and enrolled all consecutive eligible children to achieve 150 symptomatic children sampled. If they identified any skin lesion, the child was invited for diagnosis, swab sample collection and treatment after their parents or guardians provided written informed consent. Similar procedures were used for post-TCT surveys that were conducted only in the Abamkrom sub-district.

Children with suspected yaws had their lesions photographed and specimens taken directly from the largest papilloma or ulcer after cleansing with sterile saline using either a sterile plastic curette (Ear Curette, Sklar Instruments, West Chester, PA, USA) or sterile dacron-tipped swabs (Medical Wire & Equipment, Corsham, UK) for PCR testing. *PCR-confirmed yaws* was defined as a suspected case with a positive PCR detection of DNA of the polymerase I gene and/or the *T*. *pertenue*-specific 23S rRNA gene sequence on material collected from suspected lesions [24].

Scrapings from papillomata and swabs taken from the bases of skin ulcerations were expressed into 1.2 ml of Assay-Assure nucleic acid transport medium (Thermo Fisher Scientific, Waltham, MA, USA). All specimens were stored frozen at −20°C before shipping, on dry ice, to the WHO Collaborating Centre for Reference & Research in Syphilis Serology at the Centers for Disease Control in Atlanta, GA, USA. Genomic DNA was extracted from 350 μl aliquots of assay-assure samples using the iPrep PureLink gDNA blood kits (Life Technologies, Grand Island, NY, USA) and iPrep purification instrument.

The specimen DNAs were originally screened with TaqMan-based real-time 4-plex polymerase chain reaction (PCR) targeting *tp858* and two areas of the *tprl* (*tp620*) [25]. However, due to the discovery of primer binding site mutation (i.e. some strains may be undetectable by the PCR used), [26] we changed to a more sensitive PCR strategy and all specimens were re-tested using a real-time PCR targeting the DNA polymerase I gene (*polA*, *tp0105*) of pathogenic treponemes (which detects all 3 *T*. *pallidum* subspecies) [24] and a real-time 3-plex PCR that detects the two 23S rRNA point mutations (A2058G and A2059G) associated with macrolide resistance in *T*. *pallidum* described previously [27]. If a specimen tested positive by *polA*-PCR and/or 23S rRNA PCR (wild type or mutant), then we used the TaqMan-based real-time 4-plex PCR to differentiate *T*. *pallidum* spp. *pertenue* from spp. *pallidum* and *endemicum* [25]. A nested-PCR and sequencing of a portion of *tp858* were used to resolve discrepant results among those three assays and to confirm the presence of *T*. *pertenue*–specific DNA sequences.

All DNA samples were tested for *Haemophilus ducreyi* (which had previously been detected in yaws-like lesions in PNG, Ghana, Vanuatu and the Solomon Islands [28–31]) and *Mycobacterium ulcerans* (the causative bacterium of Buruli ulcer, known to be endemic in the region) using specific sequences (hemolysin gene, *HdhA*) and Insertion Sequence (IS) 2404 respectively in a real time duplex PCR [32,33]. Genomic DNA samples purified from *H*. *ducreyi* or a *M*. *ulcerans* culture (kindly provided by Dr. Anthony Ablordey, Noguchi Memorial Institute for Medical Research, University of Ghana, Legon, Ghana) were used as the positive controls for PCR assays. At least one no-template-control (NTC) and one positive control were included in every test run. The analytical sensitivity of the duplex PCR assay is approximately 10–100 copies per reaction and the analytical specificity was assessed using DNA from a panel of organisms including commensal and pathogenic microbes found in the genitourinary tract and as part of the normal skin flora. All real-time multiplex PCR assays were performed on a Rotor-Gene Q real-time PCR instrument (Qiagen Inc.,Valencia, CA, USA).

In addition, serum samples were obtained from venous blood collected from all children with active lesions, for laboratory-based testing. These were stored frozen at −20°C and transported on dry ice to the WHO Collaborating Centre for Reference & Research in Syphilis Serology, where they were tested using a quantitative rapid plasma reagin (RPR) test (Alere North America, Inc., Orlando, FL, USA) and a *T*. *pallidum* passive particle agglutination assay (TPPA, Fujirebio Diagnostics Inc., Malvern, PA, USA).

### Statistical analysis

Data were entered in Microsoft Access software, version 15.0 (Microsoft, Redmond, WA, USA) at the Ministry of Health, Ghana. The integrity of the data was verified by using a double data entry process. The primary outcome was change in prevalence of dual seropositivity following TCT, secondary outcomes were changes in rates of prevalence of suspected cases with lesions and change in prevalence of cases with lesion and seropositivity. We calculated that a sample size for the pre- and post-TCT surveys of at least 854 schoolchildren aged 5–15 years (EPI INFO 2000 sample size calculator) was required to detect a reduction by 45% in yaws seroprevalence among students before and after TCT intervention with a 95% confidence interval (CI) and 80% power. A design effect of 2 for the cluster sampling method was used to calculate the power [34,35]. The average number of children among the 24 schools of the subdistrict is 145.5, therefore we considered that at least 10 schools had to be sampled at each survey.

We calculated prevalence rates and 95% confidence intervals using the clustered sandwich estimator to control the variability of clusters. We evaluated the changes in yaws seroprevalence, and prevalence of yaws-like lesions among the schoolchildren sampled before and one year after TCT using logistic regression models controlling the variance-covariance matrix (VCE) corresponding to the parameter estimates. We reported the standard errors of parameter estimates as the square root of the variances of the VCE. For these, we use the option cluster in the calculus of Odds Ratios with the logistic regression models. We calculated Odds Ratios (post- compared to pre- TCT) for positive serology or clinical findings. The differences in the prevalence rates were considered statistically significant when two-sided p-values were less than 0.05. The statistical analysis was performed with Stata StataCorp. 2017 (Stata Statistical Software: Release 15. College Station, TX: StataCorp LLC)

## Results

### Mass treatment with azithromycin

During the 5-day community-based mass treatment campaign, 14 548 (89%) of 16,287 residents in the sub-district received a single oral dose of azithromycin. Individuals who were not eligible for treatment (977, 6.0%), or absent during the mass treatment (762, 4.7%) accounted for 10.7%. of the total population (16 287) of the sub-district. There were no severe adverse events attributable to the study drug; only 45 (0.3%) of the 14 548 participants treated reported mild to moderate self-limiting adverse events including abdominal discomfort, nausea and vomiting.

### Demographics of the surveyed schoolchildren

Of the 943 children examined at schools before the community-based mass treatment campaign, 487 (51.6%) were male, and a similar proportion of males was found among those examined at schools after the TCT (632 /1211, 52.2%). The mean (SD) age of the pre-TCT schoolchildren (10.5 [2.5] years) compared with that of the post-TCT children (9.4 [3.6] years) was not significantly different.

### Pre- and post-TCT clinical and serological results

The prevalence rate of dual seropositivity in the DPP test decreased significantly, from 103/943 (10.9% 95% CI 6.5–17.5) among children in the pre-TCT survey to 27/1211 (2.2%, 95%CI 1.3–3.7; OR 0.19, 95%CI 0.09–0.37) in the post-TCT survey (Table 1). In addition, the prevalence rate of serology confirmed yaws-like active lesions among schoolchildren was significantly reduced from a pre-TCT rate of 54/943 (5.7% 95%CI 3.2–9.9) to 7/1211 (0.6% 95%CI 0.2–1.6; OR 0.10, 95%CI 0.25–0.35) in the sample of schoolchildren examined one year following the TCT.

**Table 1:** Changes in seroprevalence, prevalence of skin lesions and serologically confirmed skin lesions among schoolchildren in the Abamkrom sub-district of Ghana, before and one year after TCT with 30 mg/kg azithromycin.

Burden of disease	Pre-TCT number (%)	95% CI	Post-TCT number (%)	95% CI	Odds Ratio (95% CI)
Yaws seroprevalence (dually-positive DPP rapid test)	103/943 (10.9%)	(6.5%-17.5%)	27/1211 (2.2%)	(1.3%-3.7%)	0.19 (0.09–0.37)
Prevalence of skin lesions	337/943 (35.7%)	(30.8%-40.9%)	223/1211 (18.4%)	(14.1%-23.6%)	0.41 (0.31–0.52)
Prevalence of yaws-like lesions with a dually positive DPP rapid test	54/943 (5.7%)	(3.2%-9.9%)	7/1211 (0.6%)	(0.2%-1.6%)	0.10 (0.25–0.35)

### Results of PCR on lesion swabs to determine etiology

The results of PCR testing obtained from the children with active lesions in the extended intervention area pre- TCT are shown in Table 2. Among 158 children with active skin lesions sampled before mass treatment, 29/158 (18.4%) tested positive for *T*. *pertenue*-specific DNA sequence (none of which contained mutations associated with azithromycin resistance) and 45/158 were *H*. *ducreyi* positive including 7/158 lesions that were dually *T*. *pertenue* and *H*. *ducreyi*-PCR positive. *M*. *ulcerans*-specific DNA sequences were not detected in any specimen obtained from lesions, either pre- or post-TCT. In the Abamkrom sub-district 3/53 (5.7%) sampled cases pre-TCT were *T*. *pertenue-*PCR positive (Table 3), compared with 0/49 (0.0%) of specimens sampled one year after mass treatment while the proportion of *H*. *ducreyi*-positive ulcers remained largely unchanged.

**Table 2:** Patterns of seroreactivity (RPR and TPPA laboratory-based tests) among children with active skin lesions consistent with yaws, by detection of *T*. *pertenue* and *H*. *ducreyi*-specific DNA sequences, pre- TCT in the entire West Akim district (8 sub-districts).

Pattern of seroreactivity	Pre-TCT			
	*T*. *pertenue*-PCR + no.+/total (%)	*T*. *pertenue- and H*. *ducreyi*-PCR + no.+/total (%)	*H*. *ducreyi*-PCR+ no.+/total (%)	PCR negative no.−/total(%)
TPPA+/RPR+	20/57 (35.1%)	6/57 (10.5%)	7/57 (12.3%)	24/57 (42.1%)
TPPA+/RPR-	1/8 (12.5%)	1/8 (12.5%)	2/8 (25.0%)	4/8 (50.0%)
TPPA−/ RPR+	0	0	0	0
TPPA−/RPR-	1/93 (1.1%)	0	29/93 (31.2%)	63/93 (67.7%)
Total	22/158 (13.9%)	7/158 (4.4%)	38/158 (24.1%)	91/158 (57.6%)

**Table 3:** Results of PCR in specimens collected in the Abamkrom sub-district.

PCR analysis	Pre-TCT	Post-TCT	OR (95% CI)
T. pertenue-PCR + no.+/total (%)	3/53 (5.7%)	0/49 (0.0%)	NA
*H*. *ducreyi*-PCR+no.+/total (%)	12/53 (22.6%)	14/49 (28.6%)	0.73 (0.27–1.96)
*M*. *ulcerans*- PCR+no.+/total (%)	0/53 (0.0%)	0/49 (0.0%)	NA
Macrolide resistant *T*. *pertenue* no.+/total (%)	0/3 (0.0%)	0/0 (0.0%)	NA
PCR negative no.+/total (%)	39/53 (73.6%)	35/49 (71.4%)	1.11 (0.42–2.91)

Of the 158 blood samples collected pre-TCT from children with active lesions that underwent serological testing at the CDC laboratory (Table 2), 57 (36.1%) showed reactivity in both the non-treponemal and treponemal tests, and 8 (5.1%) were reactive for treponemal antibody alone. Among dually reactive specimens, only 7/57 (12.3%) had RPR titres ≤1:4, while the remaining 50/57 (87.7%) sera exhibited titres between 1:8 and 1:128. The agreement between *T*. *pertenue*-PCR and serologic assays was high. However, RPR and TPPA were positive in 31 children with lesions that were *T*. *pertenue*-PCR negative (24 all PCR tests negative, 7 *H*. *ducreyi*-PCR positive; false positivity rate 31/63, 49.2%) which can be explained because serology remains detectable in serofast status.

## Discussion

Our study demonstrates that the provision of mass azithromycin administration given as a single oral dose of 30 mg/kg, up to a maximum dose of 2 g, is effective in reducing both the rates of seropositivity and the presence of serologically positive skin lesions consistent with yaws. Our results support the findings of earlier publications from PNG [17] and the Solomon Islands [18] and for the first time provides information on the efficacy of the Morges Strategy in an area that was geographically contiguous with neighboring endemic areas, unlike the previous studies. In addition, we used potentially more operationally feasible approach compared to the studies in the Pacific countries to measure the impact of one round TCT by focusing on the school-going population. However, we suggest that if this approach is used, school attendance rate should be >75% like in lymphatic filariasis surveys.

One year after TCT, we recorded a reduction in the prevalence of active yaws-like skin lesions among schoolchildren living in the targeted communities which was consistent with a reduction of passively detected suspected yaws cases in the same sub-district area (from 103 cases in 2012 to 20 in 2014) recorded in the routine reporting system (DHIMS2) of the Ministry of Health. Although the sample size was extremely small, we were unable to detect *T*. *pallidum* spp. *pertenue* using PCR test in any lesions that were clinically diagnosed as yaws seen in a survey conducted one year after TCT.

The population coverage that was achieved in the Ghanaian population reported here (89%) was slightly higher than that achieved in the PNG study (84%) [17]. The impact observed in both studies was very large consisting of a reduction by 90% of active yaws, but in Ghana we observed a lack of *T*. *pertenue-*PCR positive lesions (albeit small number of positives detected pre-TCT in the Abamkrom subdistrict) which could be related to the higher coverage rate achieved in Ghana or to the initial lower burden of infection in the present study or both when compared to PNG.

Recent studies on yaws conducted in PNG [28], Ghana [29], Vanuatu [30] and the Solomon Islands [31] have identified *H*. *ducreyi* as an important cause of skin ulcers in yaws endemic communities. Isolates of *H*. *ducreyi* obtained from skin lesions from children in Ghana are fully sensitive to azithromycin in vitro and the antibiotic is frequently used to treat chancroid, a sexually transmitted infection also caused by *H*. *ducreyi*. In our study, the overall prevalence of lesions caused by *H*. *ducreyi* was greatly reduced after TCT. However, unlike yaws, the relative proportion of *H*. *ducreyi*-positive ulcers remained essentially unchanged following TCT. It seems logical to speculate that community mass treatment with azithromycin may have less impact on lesions caused by this bacterium than those caused by *T*. *pallidum* spp. *pertenue*. We raise two possible explanations: Firstly, *H*. *ducreyi* strains that cause skin lesions in children may be more infectious than *T*. *pallidum* spp. *pertenue*. Secondly, in common with sexually transmitted *H*. *ducreyi* infections, non-sexual transmission of *H*. *ducreyi* does not appear to engender protective immunity to subsequent re-infection, unlike the transient immunity that occurs following treponemal infection [36, 37].

Our study has some limitations. First, the use of school-going children as a sampling methodology may introduce bias because the poorest children, who are at most risk of the disease, may not attend school. However, school-sampling is generally considered a good and convenient sub-population sample for other NTDs and routine surveillance data from DHIMS2 confirmed the overall decrease of yaws-like cases seen in the sub-district one year after the mass treatment. Further studies to compare the impact of interventions on school versus community-based populations are clearly indicated. Second, we selected two different groups of schools for pre- and post-TCT assessments, rather than returning to the original schools to determine the impact of TCT on the children who were seen initially. We considered that the additional survey for clinical and serological screening, and treatment of positive cases, that these schools would receive was effectively an additional public health intervention that is not a normal part of the larger interventions that the study aimed to evaluate. We therefore randomly selected a different group of schools for the post-TCT evaluation one year later to avoid measuring the potential impact of a double treatment. Third, the DPP point-of-care test that was used in this study lacks some sensitivity at low titres compared with conventional laboratory-based testing [23]. However, we believe that since the DPP test is capable of detecting non-treponemal antibody in more than 90% of cases with RPR titres ≥ 1:8 (which have previously been more closely associated with proven infectious lesions) [17] the benefits of field testing outweigh the logistics of providing a more reliable laboratory-based service which may not be readily available in impoverished yaws-endemic regions. Indeed, the lack of sensitivity of the DPP test at low titre could actually be a benefit since, in this study, confirmed low-titre RPR seropositivity (≤ 1:4), which could be missed when using the DPP test, was not associated with any lesion actively shedding *T*. *pertenue*. Finally, to determine the sample size for our surveys, we used a design effect of 2 based on previous experience in the Solomon Islands and Fiji [34,35], but which is lower than the design effect used in similar studies on NTDs [38]. In our study, the findings are still statistically strong after accounting for clustering; therefore, we believe that the design effect used was not a major limitation. However, a comprehensive review of sampling methods for yaws should be made to provide appropriate guidance for future intervention studies.

It seems clear, from the results of this intervention study, that yaws-like lesions caused by *H*. *ducreyi* or other unknown pathogens may continue to persist after a single-round of mass treatment giving the erroneous impression to both the affected population and health authorities alike that yaws has not been eliminated from a previously endemic community. This situation is compounded by the finding that *H*. *ducreyi*-positive lesions may be associated with dually positive non-treponemal and treponemal serological results in children with latent yaws or serofast status. Azithromycin is one of the antibiotics recommended for the treatment of sexually transmitted *H*. *ducreyi* infections [39,40], and it is also effective in the treatment of cutaneous ulcers in children caused by *H*. *ducreyi* [41]. In the long term, it may be necessary to devise appropriate “syndromic” management protocols for non-yaws / non-*H*. *ducreyi* lesions following the successful elimination of yaws through TCT. Further studies on the etiology of these yaws-like skin lesions in various yaws-endemic regions around the world are required.

In conclusion, our findings provide additional evidence that one round of TCT with azithromycin with high coverage ~ 90%, as part of the WHO Morges Strategy, is highly effective in providing a sustained and significant decrease in the prevalence of yaws 12 months after mass treatment from endemic communities, even if they adjoin other untreated endemic areas. Because 6-monthly resurveys using field staff may be costly, perhaps, if the initial coverage is >90%, in some places, a practical approach is to use trained village volunteers for ongoing active community surveillance and health promotion activities for yaws, especially, in the post-TCT phase similar to the experience of the guinea worm eradication programme [42]. Although our study is limited in size to allow us to make any firm recommendations regarding the ideal intervals between mass treatment and number of rounds required to interrupt transmission, we have presented some evidence to support the notion that with high coverage, a single round of TCT followed by TTT may be adequate and any additional TCT rounds to interrupt transmission may be carried out at intervals longer than 6 months. Further studies are needed to address these important issues. We also believe that the point-of-care DPP test applied to a sample of school-going children is a practical and convenient alternative to laboratory-based serological testing of a sample of the whole population to evaluate the effectiveness of yaws interventions in resource-poor settings. However, in view of the first report of macrolide resistance in yaws [43, 44], vigilance and close monitoring of cases are required. It is important that health workers to take specimens from any active skin lesions (papilloma and ulcer) of dually seropositive cases that are not healed or occur after TCT for PCR testing to definitely confirm yaws and detect any azithromycin resistance that may emerge among *T*. *pertenue* strains and to warrant the treatment of such cases with benzathine benzylpenicillin.

## Supporting information

S1 Table Deindentified data-set of the pre-TCT survey to estimate seroprevalence of yaws (Table 1). (XLSX)

S2 Table Deindentified data-set of the post-TCT survey to estimate seroprevalence of yaws (Table 1). (XLSX)

S3 Table Deindentified data-set of the pre-TCT survey to determine the etiology of skin lesions in West Akim district (Table 2). (XLSX)

S4 Table Deindentified data-set of the pre-TCT survey to determine the etiology of skin lesions in Abamkrom sub-district (Table 3). (XLSX)

S5 Table Deindentified data-set of the post-TCT survey to determine the etiology of skin lesions in Abamkrom sub-district (Table 3). (XLSX)
